# Supplementary material for: Genetic Diversity and Conservation of Bomarea ovallei (Phil.) Ravenna: Microsatellite Markers Reveal Population Vulnerability in the Atacama Desert
Source: Plants (Basel). 2025 May 14;14(10):1468. doi: 10.3390/plants14101468 (PMC12115073; doi:10.3390/plants14101468)
Supplement: Supplementary file 1 [file plants-14-01468-s001.zip › plants-3564977-supplementary.pdf]

Table S1. Characteristics of a novel set of 34 SSR loci developed for *Bomarea ovallei*. Loci highlighted in gray were fluorescently labeled and utilized in the genetic analyses conducted in this study.

Locus	5` - sequence - 3`	Accession	motiff	Size (bp)	T°a (°C)
SSRBO3554	F-AGTTTCTGACCCTCCACCTC<br>R-AAACGGTTGGTGGGAAATCA	PV126291	$(AGA)_8$	114	59
SSRBO8819	F-AGTCGAGTTTCTCTGATCTTAAA<br>R- ACAGAATCCAAAATAAAACTGGG	PV126292	$(TTA)_9$	153	59
SSRBO10325	F-ACCAAATCACTTCTACTCCAAA<br>R- ACTGGGTTTATAAGTCGGATAAA	PV126293	$(TTA)_9$	219	59
SSRBO12430	F-GAAGGTGACATGGCGTTGA<br>R- GTCAGGGACCTACATGTAATTAC	PV126294	$(TTA)_9$	118	59
SSRBO15268	F-TCAGGCAGAAACATCCATAC<br>R- ATCGCATATCAACATACATAGC	PV126295	$(TA)_{17}TGTT(GT)_{17}$	205	59
SSRBO1597	F-TGCGAGCTATGAGAAGAAAAGC<br>R- GAAGGAGAAGAGAGGAGCCC	PV126296	$(TTC)_8$	228	59
SSRBO1785	F-TGGCGTGACTCCATATGCTT<br>R- AGTGCTGCTTCGACTTCTCC	PV126297	$(TTC)_{14}$	263	59
SSRBO10757	F-ATGCCTTGTCCATGTTGTCG<br>R-CTTCCAAGGCCAAGTCGATG	PV126298	$(TTC)_6$	125	58
SSRBO11175	F-CCACATCTTGCTCCAACTCC<br>R- TCTAATCTTCCTCGTGCCCG	PV126299	$(TTC)_7$	106	58
SSRBO4175	F-TCTTGCATCTTTCGTCCCGA<br>R- TCGGTACAAGTGGTGAGCAC	PV126300	$(AGA)_6$	105	58
SSRBO16864	F-ATCATCAAAGATAGTAGCGGAAT<br>R- CTGATCTTTGTCTCTCTCCG	PV126301	$(TCA)_9$	169	59
SSRBO1032	F-TGACAAGAGCGGCAAATTCA<br>R- TGTTCCTCATTCTTTGCCTTCT	PV126302	$(TCA)_6$	281	59
SSRBO9718	F-CACAAATGCCTCACTGCTGT<br>R- TCGAGGCCAGAGACAGAAAG	PV126303	(TCA)7	126	59
SSRBO17248	F-GGTCATTCGCTGCCTACTGA<br>R- GAATTAACCGACGCGTCCTC	PV126304	$(AG)_{19}$	149	59
SSRBO17638	F-AGGGAACACAAAGAGAGGGG<br>R- CCTCTCTCTGGTCCCTCTCT	PV126305	$(AG)_{19}$	117	59
SSRBO1213	F-GCCATGTTTACACCCAGCTT<br>R- CAGAGAAGGAGGTGACGCTG	PV126306	$(AG)_{12}$	142	59
SSRBO1806	F-CGACACTGTTTTCCCTAAAG<br>R- CTTATGCTCAGCCCTTATTTC	PV126307	$(AG)_{23}$	287	59
SSRBO2008	F-CAAGTACTGAAGATCGGACAAGG<br>R- CCCTTGTTGAGAGACCAATCC	PV126308	$(AG)_{24}$	141	59
SSRBO6304	F-TGTAGCCGCCATTAGAAGGG<br>R- CAGAGAGAGGTCAGGGAGC	PV126309	$(AG)_{13}$	103	58
SSRBO14910	F-GGGACTCCACCATACTCCAC<br>R- CCACACCTTATGCCCCAAAC	PV126310	$(AG)_{15}$	150	58
SSRBO15934	F-AGAGAGAGTTGAGAGAGCTTTCA<br>R- CCTCCTTCAACGCACTCAAG	PV126311	$(AG)_{13}$	117	59
SSRBO17567	F-TGTATTTATGTCCCGTGGCA<br>R- TCTAAGATGGTGTAATTGGAGGA	PV126312	$(CT)_9TT(CT)_{15}$	204	58
SSRBO3052	F-CCTGCGAAGTCACCAATGTC<br>R- CAAGAATGCAATCAAAACCGTGA	PV126313	$(CT)_{13}$	172	59
SSRBO4022	F-ACGCGACAATACGGTTTCAA<br>R- TCGAACACTATAATGCGAAGAGT	PV126314	$(CT)_{13}$	280	58
SSRBO9629	F-CCCTTTTATGGTTTGTTGTAGCA<br>R- AGCCAAGAACAAACCGTAAAG	PV126315	$(CT)_{21}$	195	59
SSRBO10840	F-CCTTCCTCCTCTCAATCCTCTC<br>R- GCAAGAGAGAGTGGGGAGAT	PV126316	$(CT)_{21}$	151	58
SSRBO14256	F-GGTCTTAGGGTGTGGGCTAA<br>R- GTGGCATGGAAAGGAACACA	PV126317	$(CT)_{14}$	169	59
SSRBO15462	F-ACACAGTTCTAGGACAAGGCA<br>R- TTCCACAAGCCACCTTCTCT	PV126318	$(CT)_{15}TT(CT)_{11}$	109	59
SSRBO29	F-TGTTCAATCAAGTCATGGGCA<br>R- CTGGTGTAGTATCATGCATGCA	PV126319	$(TGA)_7$	204	59
SSRBO3955	F-TCGCACTTTCTCTCTGTCCT<br>R- AAACTCTATCTAAACATCCGCCA	PV126320	$(TGA)_6$	232	59
SSRBO1064	F-TGAGTTGCTGTGGTGGTATG<br>R- TCACCAAACAACCATAACACCA	PV126321	$(TTG)_{11}$	105	57
SSRBO1491	F-GAGTGCCTTGATCTCTTTGTCC<br>R- TGCTTCTCAATGCCCGAGTA	PV126322	$(TTC)_6$	250	58
SSRBO4536	F-CTCTATTTGACCCTAGCTCCCA<br>R-AGGTTGAGTGTAGTGTGAGGA	PV126323	$(AAC)_5$	159	58
SSRBO20205	F-TCAGGCGATTGGTTGGAAAG<br>R-GTTTGGGACGCGGTTTCTTT	PV126324	$(TTC)_7$	118	59
